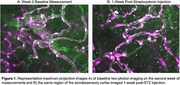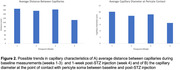# Serial Imaging of Murine Brain Capillary Pericyte Dynamics in Hyperglycemia

**DOI:** 10.1002/alz.088208

**Published:** 2025-01-03

**Authors:** Kareem El‐Ghazawi, Ukpong Eyo, Shayn Peirce‐Cottler

**Affiliations:** ^1^ University of Virginia, Charlottesville, VA USA; ^2^ University of Virginia School of Medicine, Charlottesville, VA USA

## Abstract

**Background:**

The microvasculature of the central nervous system (CNS), which delivers oxygen and nutrients and forms a critical barrier protecting the CNS, is deleteriously affected by both Alzheimer’s Disease (AD) and Type 2 Diabetes (T2D). Previous studies have shown pericyte dropout and vessel constriction in brain capillaries in AD, while other studies have shown pericyte bridging and dropout in retinal capillaries in T2D. T2D patients have increased risk of AD, suggesting potentially related microvascular pathological mechanisms. Pericytes are an ideal cell type to study for functional links between AD and T2D. The purpose of this study was to evaluate the effects of T2D on the dynamics of brain capillaries and pericytes.

**Method:**

*Myh11*‐CreER^T2^ ROSA floxed STOP tdTomato mice were fed a soft tamoxifen chow for 2 weeks. Cranial window surgery was performed in anesthetized mice, and mice underwent weekly serial live two‐photon imaging with retro‐orbitally injected FITC‐Dextran throughout a 3‐week period. Streptozotocin (STZ) (200mg/kg) was injected intraperitoneally, and mice were imaged 1‐week post‐injection. Z‐stack projections of brain tissue from the surface to 100mm deep (442um^2^ field of view) were collected, and capillary orientation and pericyte morphology were compared across timepoints and before and after STZ treatment.

**Result:**

Quantitative assessment revealed that capillaries move closer to each other 1 week after STZ injection, as indicated by a 30% reduction in the average distance between capillaries. Moreover, wherever capillaries were in contact with pericyte soma, capillary diameter was reduced by 31% post‐STZ injection compared to pre‐STZ timepoints, suggesting a constrictive role of pericytes in a hyperglycemic environment.

**Conclusion:**

Our study demonstrates how live, serial two‐photon imaging of the same fields of view of the mouse cortex can reveal dynamic capillary and pericyte morphological changes in response to a hyperglycemic environment. This experimental model system can be deployed to investigate how pericytes changes over time may link microvascular changes in T2D to those in AD. Future studies will include additional analysis time points, as well as studies in 5xFAD mice, a widely used model of AD.